# Data Representation Structure to Support Clinical Decision-Making in the Pediatric Intensive Care Unit: Interview Study and Preliminary Decision Support Interface Design

**DOI:** 10.2196/49497

**Published:** 2024-02-01

**Authors:** Najia Yakob, Sandrine Laliberté, Philippe Doyon-Poulin, Philippe Jouvet, Rita Noumeir

**Affiliations:** 1 École de technologie supérieure Montreal, QC Canada; 2 Polytechnique Montreal, QC Canada; 3 Pediatric Intensive Care Unit, CHU Sainte-Justine Montreal, QC Canada

**Keywords:** data representation, decision support, critical care, clinical workflow, clinical decision-making, prototype, design, intensive care unit

## Abstract

**Background:**

Clinical decision-making is a complex cognitive process that relies on the interpretation of a large variety of data from different sources and involves the use of knowledge bases and scientific recommendations. The representation of clinical data plays a key role in the speed and efficiency of its interpretation. In addition, the increasing use of clinical decision support systems (CDSSs) provides assistance to clinicians in their practice, allowing them to improve patient outcomes. In the pediatric intensive care unit (PICU), clinicians must process high volumes of data and deal with ever-growing workloads. As they use multiple systems daily to assess patients’ status and to adjust the health care plan, including electronic health records (EHR), clinical systems (eg, laboratory, imaging and pharmacy), and connected devices (eg, bedside monitors, mechanical ventilators, intravenous pumps, and syringes), clinicians rely mostly on their judgment and ability to trace relevant data for decision-making. In these circumstances, the lack of optimal data structure and adapted visual representation hinder clinician’s cognitive processes and clinical decision-making skills.

**Objective:**

In this study, we designed a prototype to optimize the representation of clinical data collected from existing sources (eg, EHR, clinical systems, and devices) via a structure that supports the integration of a home-developed CDSS in the PICU. This study was based on analyzing end user needs and their clinical workflow.

**Methods:**

First, we observed clinical activities in a PICU to secure a better understanding of the workflow in terms of staff tasks and their use of EHR on a typical work shift. Second, we conducted interviews with 11 clinicians from different staff categories (eg, intensivists, fellows, nurses, and nurse practitioners) to compile their needs for decision support. Third, we structured the data to design a prototype that illustrates the proposed representation. We used a brain injury care scenario to validate the relevance of integrated data and the utility of main functionalities in a clinical context. Fourth, we held design meetings with 5 clinicians to present, revise, and adapt the prototype to meet their needs.

**Results:**

We created a structure with 3 levels of abstraction—unit level, patient level, and system level—to optimize clinical data representation and display for efficient patient assessment and to provide a flexible platform to host the internally developed CDSS. Subsequently, we designed a preliminary prototype based on this structure.

**Conclusions:**

The data representation structure allows prioritizing patients via criticality indicators, assessing their conditions using a personalized dashboard, and monitoring their courses based on the evolution of clinical values. Further research is required to define and model the concepts of criticality, problem recognition, and evolution. Furthermore, feasibility tests will be conducted to ensure user satisfaction.

## Introduction

### Background

In the pediatric intensive care unit (PICU), clinicians are required to make clinical decisions daily regarding inpatients’ health conditions. In critical care, data access accuracy and speed are crucial for optimizing the decision-making process. However, the following are some factors that can limit the effectiveness of this decision-making process: (1) clinicians must deal with a high volume of clinical data from several sources, such as physiological monitors, laboratory systems, and caregiver notes on the electronic health record (EHR) [1], which can lead to delays in processing data and reaching a decision; (2) decisions made in intensive care units rely on clinical judgment based on the clinician’s knowledge and experience, which are variable [2]; (3) clinical uncertainty in critical care and the variability of cases may lead to inconclusive decisions [3]; and (4) clinician’s stress, lack of sleep, and multiple stimuli can interfere with decision-making.

To gather relevant information, clinicians have to go through several systems, browsing through different sections of an EHR, laboratory systems, and imaging systems. They must sort and analyze all this information based on their personal expertise and available scientific evidence before making any decision regarding patient care.

For the last few decades, the emergence of clinical decision support systems (CDSSs) has assisted clinicians in their cognitive process by combining scientific knowledge bases with patient data for personalized and adapted management [4]. The design of the CDSS can take different forms depending on the clinical needs [5]. These systems focus on specific problems in the environment where they are to be implemented and often rely on existing systems and organizational contexts [6]. Therefore, understanding the workflow of existing systems is essential for supporting the adoption and optimal use of a new system. This understanding of the existing systems helps to determine when and how CDSS will be used [7].

To develop decision support mechanisms, participative approaches have been used to optimize the representation of clinical data. Faiola et al [8] adopted a human-centered approach to design a decision support tool, which has been shown to be effective in reducing the cognitive overload experienced by users [8]. More design and integration approaches have been developed based on domain-specific characteristics and matching users’ cognitive processes [9,10]. Clinical data display optimization, including EHRs focused on patient-centered care [11] and dedicated clinical decision support tools that depend on specialized knowledge bases [12], has attracted the interest of other researchers.

End user involvement is essential to ensure optimal data representation, which can be achieved through observation of clinical activities, individual interviews, and focus groups [13]. Data visualization must be validated by clinicians to ensure that it is understandable, relevant, useful, and readily available [14,15].

In this study, we aimed to adapt the data representation structure to the clinical processes in the PICU and allow its application in various clinical care scenarios. Although most decision support research in the literature focuses on specific clinical needs, the objective of our study is to facilitate the integration of multiple CDSSs developed for specific problems and the use of such systems for specific patients while ensuring harmonized monitoring and adequate evaluation for all hospitalized patients.

Our approach involved the end users throughout the data representation implementation process, including needs identification and prototype design, to illustrate the targeted structure.

### Literature Review

#### Clinical Decision-Making

The clinical decision-making process is based on 2 main approaches: an intuitive heuristic approach, which is triggered in uncertain or critical situations requiring rapid intervention, and an analytical approach that involves gathering and processing information before reaching a conclusive decision. In clinical practice, the decision-making process varies with the clinician’s experience, their developed cognitive model, and processed information [16]. Furthermore, to refine clinical decisions and reduce the risk of errors, clinicians rely on knowledge bases and scientific evidence to process patient-specific data [17]. This adds complexity to the cognitive process in terms of time and effort invested.

In critical care, clinical teams typically discuss patients’ status and care during handoff meetings and medical rounds. Decision-making at these times depends on the relevance and accuracy of the data presented [18]. Decision support mechanisms are increasingly integrated into clinical processes to reduce the information gap by making relevant knowledge and data readily available through computerized systems.

#### Use of CDSSs

CDSSs are computer-based solutions that support clinicians and health care professionals in making clinical decisions [19] by providing them with person- or population-specific knowledge and information. This information is filtered and presented in a convenient timeframe to improve the health care of individuals and promote better population health [20].

Historically, CDSSs have been used for preventive, diagnostic, and therapeutic purposes, with the primary goal of improving the quality, safety, and efficiency of patient care [21]. Depending on the context of use, these systems may include best-practice guidelines for specific conditions or suggestions based on patient clinical data [22].

A CDSS is usually supported by an inference engine that incorporates clinical practice guidelines with patient-specific data to generate tailored suggestions [4]. However, other models are increasingly used, and artificial intelligence is used to predict condition changes or deterioration [23]. Computerized systems encompass 5 common types of decision support methods for knowledge sharing to reduce the risk of error among clinicians: order sets, information buttons, data documentation forms and templates, alerts and reminders, and relevant data representation [24].

The synthetic representation of patient data is a major challenge, mainly because of variability in data sources and format, along with the integration of medical knowledge in data processing. To implement such a representation, researchers have developed integration and structure design approaches that rely on the specificities of the work domain and adapt to users’ cognitive processes [9,10].

Improved visual representation facilitates timely information access, which has a positive impact on clinicians’ performance and cognitive processes [25,26]. Therefore, selecting adequate, reliable, and relevant content and using simple and understandable messages is highly recommended. Furthermore, clinicians’ time must be optimized by providing accurate and timely information and avoiding double entries by ensuring interoperability with EHR [27]. Finally, incorporating these systems into the users’ workflow is key to optimizing their implementation [28].

Wright et al [29] developed a taxonomy of clinical decision support tools to help categorize and compare their capabilities (eg, guidelines, notification, and order edition) in both commercially available and internally developed systems [28,29]. They found that a home-developed CDSS is more likely to achieve its goals as it focuses on local needs. Few studies have been conducted on implementing CDS components in commercial EHRs [30]. A review of 9 commercial systems found variability in decision support capabilities, which shows a significant gap between vendors [31]. Most EHRs focus on patient care and limit the scope of integrated CDS components to medication safety and managing lists of patients with common characteristics. However, standalone CDSSs are continuously evolving [32]. Recent work has demonstrated the feasibility of developing a flexible platform for hosting CDSS outside a specific EHR. The authors estimated that an EHR-agnostic approach facilitated the modification and development of new features because it implies fewer technical challenges [33].

The implementation of new technologies in the PICU settings should be performed carefully. This applies to CDSSs as their potential to improve clinical outcomes depends on how they are implemented in terms of integration into clinical workflow; process fluidity; interoperability and communication with the existing clinical systems; and data collection, analysis, and display. A previous study reported that commercial EHRs lacked features required in pediatric settings and that CDSSs were mostly integrated using home-developed tools in the unit [34].

At Sainte-Justine Hospital, several research initiatives have been undertaken in the PICU to develop CDSSs for specific needs, such as assistance in the automated diagnosis of acute respiratory distress syndrome in children based on various physiological and radiological criteria [35,36], assessment of the quality of head injury care in adherence to clinical practice guidelines [37], early detection of ventilator-associated pneumonia [38], and hypoxemia diagnosis and management [39]. Unlike the commercially available CDSSs, these tools developed at the Sainte-Justine Hospital were based on local clinical needs, adapted to patient characteristics in the PICU, and developed in harmony with the existing infrastructure, including devices, data availability, and access. Using additional tools to address individual problems can cause an excessive burden on clinicians. The integration of these initiatives into a unified structure will benefit both the clinical workflow through centralized information and the patient’s overall care, as each CDSS improves accuracy by targeting specific criteria.

### Research Objective

The purpose of this study was to collect and analyze clinicians’ needs in an academic hospital PICU in support of clinical decision-making and establish a data representation structure for easy and quick access to relevant information required for clinical care, depending on patients’ care trajectory. Our goal was to provide a customizable visual tool allowing an overview of the patient's data depending on their health condition (eg, diagnosis, current problem, and deterioration of the human body system) to reduce information processing time and mental overload for clinicians. This tool serves as a platform for integrating CDSSs in the PICU in response to patients’ specific needs while ensuring that the clinical flow is respected. This was the first step in implementing multimodal real-time CDSSs.

This study was not intended to replace existing clinical tools (eg, EHR, laboratory systems, bedside monitors, and ventilators) because these tools remain essential sources for acquired data and form integral parts of the intensive care unit environment. The EHR represents the core of this technological ecosystem, as it covers the patient’s trajectory from admission until being transferred or discharged. During this time, clinicians (eg, intensivists, nurses, external specialists, pharmacists, and health professionals) use the EHR’s functionalities for different purposes (eg, notes, prescriptions, reports, consultations, patient assessment, and monitoring) and have access to some decision support features, such as alerts for abnormal clinical values, task reminders, prescription aid, and events notification. Although these features help clinicians in their daily work, they do not provide further assistance in specific situations or for variable diagnoses.

In addition, we believe that an independent decision support tool allows for continuous improvement and adjustment while considering local needs. To this end, we encouraged the clinicians’ involvement throughout the study.

## Methods

### Overview

In our approach to implementing the new CDSS structure, we opted for the standard process of implementing computerized systems, which starts with identifying end users’ needs before beginning the modeling and prototyping phase and then continues with performing tests to finally allow its integration into the clinical flow [40].

Our work focuses specifically on the first 2 phases of the process: identifying requirements through observation activities and interviews, followed by modeling and prototyping using design meetings. User testing will be covered in future work.

### Needs Identification

To identify clinicians’ needs in terms of decision support, we first participated in a day of routine clinical activities at the Sainte-Justine Hospital PICU to understand the general workflow by observing interactions between team members and how they used clinical systems.

Following our observations, we planned interviews with PICU clinicians to understand their workflow and collect data on their needs. We approached the main categories of clinical staff in the unit, including intensivists, fellows, residents, nurses, and nurse practitioners. To achieve the target sample level (>10 participants), we used different communication channels for recruitment, namely email invitations, announcements in weekly journals, and direct contact in the unit.

We enrolled 11 clinicians, including 5 intensivists, 1 fellow, 4 nurses, and 1 nurse practitioner. Semistructured interviews lasting between 30 and 60 minutes were conducted face-to-face or remotely via a videoconferencing platform based on the participants’ preferences and availability. The interviews were recorded and transcribed by the research team. The interview guide was designed to provide an understanding of the use of existing work systems, evaluate participants’ knowledge and familiarity with decision support systems, and identify their needs and expectations regarding CDSS implementation.

### Modeling and Prototyping

On the basis of data collected from the observation activities and interviews, we defined a 3-level data representation structure (ie, unit, patient, and system). This provided us with a basis for designing the first prototype. To validate the understanding of this first prototype and the relevance of the integrated functions, we held design meetings with the enrolled participants via videoconferencing. Before these meetings, the participants received a short video explanation with an evaluation survey to introduce the general functioning of the prototype and obtain their initial feedback. Our goal was to engage in interactive discussions with participants during the design meetings. To this end, we used a clinical scenario involving a patient with a severe head injury, and then we asked the participants to perform some tasks, such as sorting the patient list and assessing the patient’s health condition based on the presented data, to use the functionalities available on the prototype and to describe their understanding. Simultaneously, the participants were given the opportunity to suggest improvements for adding, removing, or correcting the represented data. A total of 5 intensivists participated in these design meetings. Depending on their availability, 3 physicians were met individually, and 2 were brought together in the same meeting.

### Ethical Considerations

The Centre Hospitalier Universitaire Sainte-Justine Ethics Review Board approved this study (CER-2022-4083), and all participants signed an informed consent form before participating in the study. Consent was obtained in person, either on the first contact or the day of the interview, after receiving a positive response to the mail invitation. All original consent forms were archived at the Sainte-Justine Research Center.

Participants’ personal information (eg, name and email) was saved separately from the study data in a password-protected Excel (Microsoft Corp) file. Personal information was linked to study data using a code for each participant. The data will be kept in a secure directory in the hospital server for 7 years, after which it will be destroyed.

No personal information was used during interviews. Only the participant codes were mentioned at the beginning of the interviews. The recordings were immediately deposited in the secure directory at the end of each interview. Once listened to and transcribed, this file was saved in another folder in the same secure directory.

A CAD $10 (US $7.5) gift card was offered to participants as a gesture of appreciation for their participation.

## Results

This section presents the findings from the data collection and analysis as well as the prototype designed to illustrate the proposed structure for clinical data representation.

### Description of the Existing Process

The observation activities allowed us to understand the clinical workflow related to team members’ interactions and how they used the existing clinical systems.

#### Clinical Workflow

##### Overview

**Figure 1:**
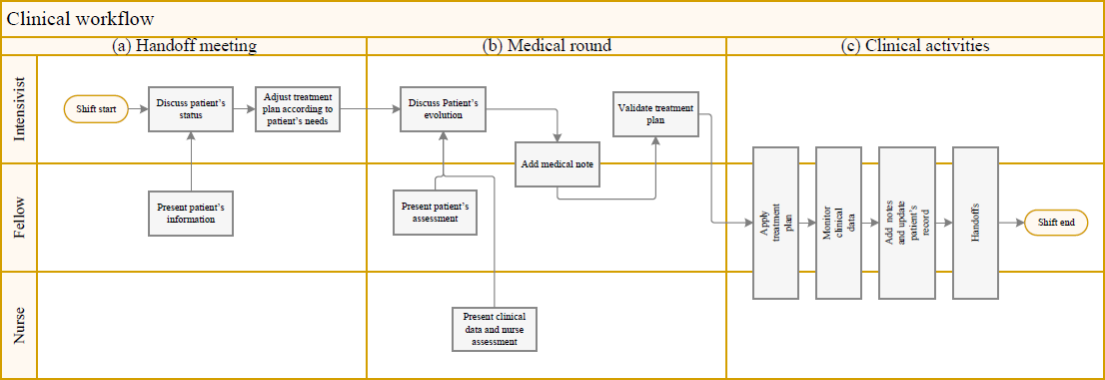
Clinicians workflow during a typical daily shift in the pediatric intensive care unit at Sainte-Justine hospital.

Figure 1 presents a typical day at the PICU. The day usually began with a handoff meeting (1) between the last medical team and the team taking over during the day, followed by a bedside visit (2) to discuss and validate the patient’s treatment plan. Subsequently, team members performed clinical interventions (3) related to their specific roles and responsibilities before handing over patient information to the next team.

We took time to observe some clinical activities, such as patient information transfer meetings and morning medical rounds.

##### Handoff Meeting

This meeting brought together pediatric intensivists or patrons, and fellows from 3 specialties: general acute pediatrics, called Pediatrics A; chronic pediatrics, Pediatrics B; and cardiac surgery, Pediatrics C. The goal was to assess the medical conditions and illness evolution of inpatients and new admissions to establish a treatment plan for the next 24 hours. Generally, patients were presented, starting with discharged patients, followed by critical or extremely ill patients, and then stable patients. For each patient, a predefined plan covered the body’s systems, including respiratory, cardiovascular, neurological, gastrointestinal, hematologic, immunologic, renal, and metabolic systems, as well as the infectious process, tegument, and musculoskeletal system. Patients were also assessed psychosocially before the medical team concluded the global assessment by proposing a treatment plan.

##### Medical Round

After the handoff meeting, the team began a collaborative round at the patient’s bedside to discuss the patient’s current condition with the nurse in charge. Parents could participate in discussions to complete the information and ask about their children’s condition. Once the discussion was completed, a patient status summary was presented with a proposed treatment plan, including new laboratory or imaging orders, medication adjustments, outpatient referrals, and other diagnostic or therapeutic interventions as needed. Once the plan was approved, a medical team member recorded the assessment summary by creating a new medical progress note in the patient’s record. This note included important laboratory results, vital signs, ventilation, the patient’s global evolution in the unit, and their evolution within the human body systems. For example, the *neurological level* included sedation and comfort assessment data, whereas the *respiratory level* included ventilatory parameters assessment and likely respiratory distress signs.

##### Clinical Activities

After the medical round, clinicians were responsible for executing the patient treatment plan and completing the associated tasks according to their profile and skills. They frequently referred to patient records to review collected clinical data, including nurses’ observations, prescriptions, laboratory results, and notes provided by external consultants such as medical specialists and health professionals (eg, respiratory therapists, physiotherapists, nutritionists, and social workers). In addition, they could access the laboratory and medical imaging systems to analyze detailed examination results.

Clinicians must document all interventions in their clinical notes on the EHR. Clinical notes were entered in free text, which meant that the information structure and volume and the terminologies and expressions used differed among clinicians. Textbox 1 illustrates the variability in the medical progress notes taken while assessing patients with respiratory problems.

**Examples of respiratory assessment in medical progress notes from electronic health records in the pediatric intensive care unit at Sainte-Justine Hospital illustrating the formatting variability among clinicians.**

**Note 1**

- #Decadran BID (0.6 mg/kg/day) last dose for the day
- Extubation 28/04 AM
- AA
- Minimal desaturation, spontaneous resolution overnight
- Bilateral GAE, no added noise, eupneic
- Venous gas 7.37/47/25
- Last RPL 28/04 improvement

**Note 2**

- # HFNC 20 LPM FiO2 40%
- Sat 90-92% More obstruction than usual
- RR 25-30 no drawing
- Secretory + physio in progress during passage
- GAE bilaterally
- Noise transmitted bilaterally
- No labs

**Note 3**

- #Ventolin IV 3 mcg/kg/min * 7h45 this morning
- #Solumedrol 1 mg/kg q6h
- # Ketamine infusion 0.5
- BiPAP Ai 5 / Peep 8 / FiO2 21%
- Reduced EA, but improving, absence of wheezing
- Indrawing
- 7.4/34/20.8

#### Use of Existing Systems

While observing the clinical activities in the PICU, we learned about the main working tools in the unit. We mainly targeted the TVL (*tableau de visualisation de lits* [beds visualization table]) unit dashboard and EHR.

##### TVL Unit Dashboard

**Figure 2:**
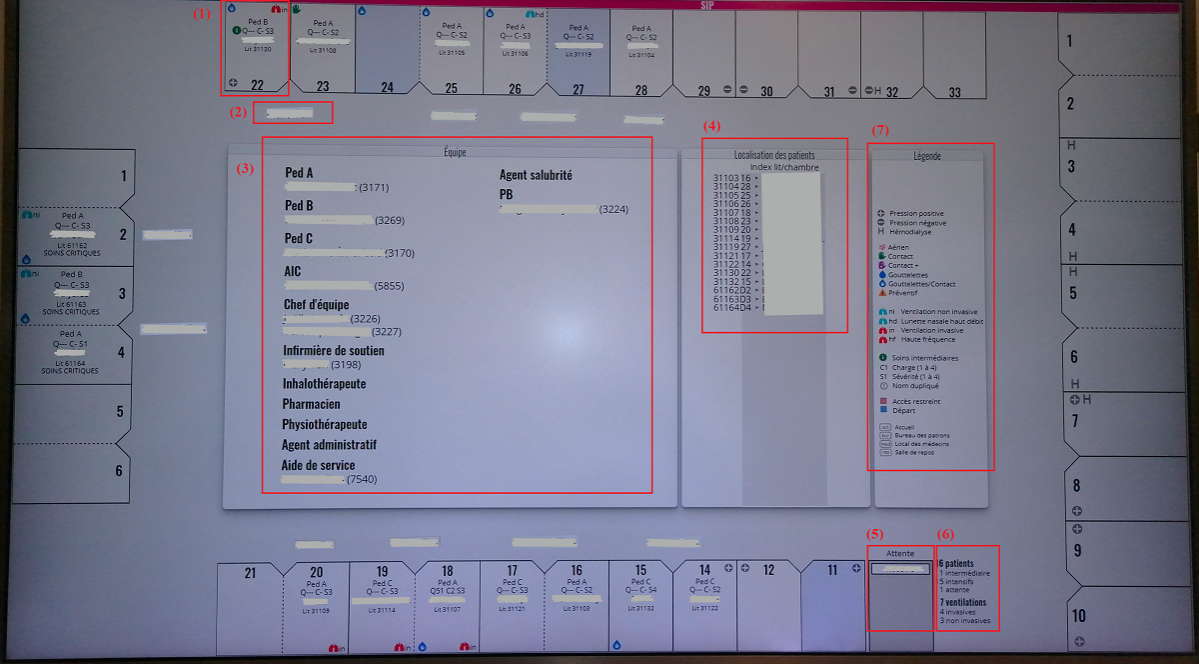
Tableau de visualisation de lits (beds visualization table; TVL) unit dashboard displayed at the pediatric intensive care unit (PICU) entry (names are hidden), which presents (1) patient room and information, (2) bedside nurse allocation, (3) the PICU team in charge, (4) room index, (5) waiting patients, (6) patients summary, (7) and the legends.

TVL is a digital display tool developed in the PICU to evaluate the unit’s capacity to receive patients and the nurses’ workloads. Besides allowing all professionals and families to easily locate a patient, as it is displayed on a large screen at the unit’s entrance, the TVL allows PICU staff to view the patient distribution, depending on the team in charge (Pediatrics A, B, and C), and identify discharged patients and new admissions [41,42]. The tool is based on an architectural representation of the units (Figure 2). It mostly contains unit management information, including (1) the patient room or bed, (2) bedside nurse allocation, (3) the team in charge, (4) room index, (5) waiting patients, (6) PICU patients summary, and (7) the legend indicating the meanings of icons (eg, room, workload, and equipment indications). The TVL contains limited information about the patient’s condition, such as ventilatory mode and circulatory support equipment, which limits its use in clinical care.

##### EHR Tool

Patient records in the PICU were managed using a dedicated critical care system known as IntelliSpace Critical Care and Anesthesia (Philips Healthcare). This system is connected to administrative modules to manage patient admissions, transfers, and discharges. It is also connected to physiological monitors for vital signs, mechanical ventilators for respiratory parameters, intravenous pumps and syringes for drug perfusion and feeding data, the pharmacy for medication prescription management, and laboratory modules for biological examination prescriptions [43]. System interoperability consolidates all clinical data from the connected systems (eg, physiological monitors, intravenous pumps, ventilator, laboratory, and pharmacy systems) into the EHR along with free text clinical notes typed by the clinicians. However, clinicians must search several sections, gather information, and analyze it to assess the patient’s condition and adjust the treatment plan. Therefore, a synthetic representation of patient data is required to guide clinicians, limit cognitive overload, and optimize the time spent collecting information relevant to decision-making.

The EHR is an integral tool and reference for clinicians in the PICU, which is used as a source of clinical data collected continuously from the patient’s environment (eg, bedside monitors and ventilators) and data collected from punctual or recurrent interventions (eg, laboratory examinations). The EHR is also used for data entry purposes to document the patient’s assessment and to add some measured values (eg, Glasgow Coma Scale and Comfort Scale scoring). Although the EHR played an essential role in the clinical workflow, the real challenge remained in the clinician’s ability to trace the required data and process it in due course [44]. Therefore, the development of a visual tool provided a targeted view of the EHR’s content without additional entry tasks for the clinicians.

The first observation phase raised our awareness of the importance of optimizing data representation to support clinicians in patient care. Considering that the EHR was the main clinical tool used in the PICU daily workflow for physicians and nurses and that it gathered data from different sources, we focused more on the information-seeking process in the EHR among clinicians and the potential use of decision support tools in their practice.

### Data Analysis

Interviews with participants provided insights into the information-seeking process through existing systems and allowed discussions about decision support systems in terms of familiarity with and clinicians’ expectations of such systems.

#### Information-Seeking

Table 1 presents the use patterns of clinical systems among participants. To gather information for decision-making, clinicians browsed through different sections in the EHR.

**Table 1:** Data sources within the existing systems and their use by PICU^a^ clinicians.

Data source		Use by participant category			
		Physician	Fellow	Nurse practitioner	Nurse
**EHR^b^**					
	Clinical data	High^c^	High	High	High
	Vital signs trends	High	High	High	High
	Prescriptions and medication	High	High	High	High
	Scores	High	High	High	High
	Admission notes	Low^d^	Low	Low	Low
	Medical progress notes	Medium^e^	Medium	Medium	Low
	Brief notes	High	High	Medium	Low
	Consultants’ notes	High	High	High	High
Laboratory system		High	High	High	High
Imaging system		High	High	Medium	Low
Clinical practice guideline		High	High	High	Medium
TVL^f^ unit dashboard		Medium	Medium	Medium	Low^g^

^a^PICU: pediatric intensive care unit.

^b^EHR: electronic health record.

^c^High: >3 times per shift.

^d^Low: 0 to 1 time per shift.

^e^Medium: 2 to 3 times per shift.

^f^TVL: tableau de visualisation de lits (beds visualization table).

^g^The frequency becomes high when the nurse is assigned a team leader.

Clinical data from physiological monitors, laboratory systems, and intravenous pumps were categorized by body systems. Data were displayed in a set of detailed tables containing values for each category and the results of nursing observations. Clinicians must navigate all the tables to find relevant information for patient assessment. Data were collected in the same way regardless of the patient’s problem, which made it difficult to analyze and process. Considering a case of brain injury, the clinician examined the clinical indicators, including biological examinations obtained from the laboratory system and physiological parameters collected from connected devices such as ventilators and feeding pumps. These indicators were associated with the patient’s condition by going through data categories (eg, neurological, respiratory, and cardiovascular) and then refined information to obtain a synthesis to support their decision, which took time. The EHR also displayed vital signs trends for a certain period. The vital signs were fed directly from the bedside monitors which were connected to the patient. Notably, some clinicians believed that trends could be improved by facilitating access to the graphs when analyzing patient data and by ensuring that abnormal values were quickly detected. Regarding prescriptions, a dedicated section allowed the display detailed drug information, such as doses and administration modalities, and tracked current prescriptions or added new ones. Furthermore, the ongoing perfusion and the drug boluses could be tracked in the EHR. The nurses collected the scores and measurements important for patient assessment and entered them in the EHR (eg, the Comfort-Behavior Scale score for intubated patient assessment and delirium and Richmond Agitation Sedation Scale score for neurological assessment).

Regarding clinical notes, their use varied based on need. Admission notes describing a patient’s illness and past medical history were generally viewed when the patient was newly admitted but continued to be important as a reference point for patient outcomes during their stay. Medical progress notes were completed daily by the medical team in charge. Data were entered in free text to describe the patient’s evolution before concluding with a treatment plan. Information entry was redundant and unstructured, which complicated its processing. To monitor patient progress in these notes, clinicians often relied on the conclusion and might also rely on brief notes to learn about reassessments made during the day. External consultant notes entered by other medical specialists and health professionals were displayed in chronological order, allowing clinicians to track them by date. However, clinicians were not notified when notes were added or modified and could not use filters to facilitate searches. This meant that PICU clinicians must repeatedly check the external consultants’ sections for new updates. In addition, they must scroll through the chronological list and search through involved specialties to locate the required note.

Although the EHR gathered the necessary data for patient care, clinicians commonly used laboratory and imaging systems for a complete examination of the test results.

Regarding the TVL dashboard, clinicians used it mostly when starting their work shift to track patients and verify who was in charge (eg, medical team and bedside nurse). Some physicians used a printed version to organize their daily schedule by taking notes directly on paper, whereas a nurse would use it, especially when assigned as a team leader, to manage the workload and resource allocation. Most of the information integrated into the TVL was not helpful in the clinical care context because it was dedicated to bed management. However, clinicians used it to help plan medical rounds.

#### Decision Support: Expectations and Needs

##### Overview

The interviews conducted enabled us to assess participants’ familiarity with the CDSS and determine their expectations with respect to these systems. In Table 2, which presents the main results, it is notable that most clinicians interviewed (8/11, 73%) reported being unfamiliar with the CDSS. Clinicians’ practice experience had no impact on their level of familiarity with the CDSS. An experienced clinician does not necessarily have specific knowledge about the CDSS or its potential use in clinical practice. Among physician intensivists, those with strong knowledge related it to their involvement in research to develop clinical decision support tools. However, we found that even clinicians with little knowledge about CDSS operations could express their expectations and needs, both for their professional development and for the benefit of their patients. For clinicians, using the CDSS would optimize their cognitive decision-making process, facilitate daily work planning and managing information flow during the busiest periods, improve clinical tools efficiency, and reduce the risk of errors and oversights by providing timely and easy access to relevant data. In addition, the CDSS could promote coaching for medical and nursing interns and support newly hired staff members. Regarding patients, the CDSS helped to improve clinical care by personalizing data processing based on the patient’s physiological and pathological characteristics while adhering to scientific recommendations and clinical practice guidelines.

**Table 2:** Participants’ experience and expectations from a decision support system to be used in PICU^a^ (N=11).

Category	Physician (n=5)	Fellow (n=1)	Nurse practitioner (n=1)	Nurse (n=4)
Years of practice (years)	7-32	6	1.5	7-25
Familiarity with the CDSS^b^	40% strong knowledge 20% medium 40% low	100% low	100% low	100% low
Expected outcomes	Guide cognitive process for decision-making Optimize daily work planning Support students in their learning process Personalize patient care management Optimize rare disease management	Reduce mental overload during busy workdays and agitated nights	Reduce the risk of error and omission Harmonize access to knowledge and data	Coach newly hired staff members Optimize use of work tools and systems
Considerations	Have a user-friendly design. Use for guided decisions. Respect clinical workflow.	Avoid intrusive alerts	Opt for simplicity and ease of use	Avoid double data entry

^a^PICU: pediatric intensive care unit.

^b^CDSS: clinical decision support system.

To ensure the efficient and successful implementation of a CDSS in their workflow, clinicians insisted on the usability and simplicity of design features while avoiding irritating factors, such as duplication of existing data entry and disruption with highly intrusive alerts. The CDSS must also fit into the users’ workflow and contribute to decisions guided and supported by clinical judgment. This meant that a clinician might find that the guidance or recommendations generated by the CDSS did not align with their conclusions based on prior knowledge and experience. In this case, if the clinician chooses to ignore the CDSS guidance, they must justify the final decision.

On the basis of the collected data, we identified 5 main themes related to clinical decision support needs (Table 3). Furthermore, we highlighted the main objectives and the means to respond to them.

**Table 3:** Clinician needs for decision support capabilities in the PICU^a^.

Themes	Needs expressed by participant category			
	Physicians	Fellows	Nurse practitioners	Nurses
Patient prioritization	Provide stability indexes Categorize patients according to their condition severity	Identify critically ill patients Notify changes in patient’s condition	Quickly detect abnormal changes in patient’s status	—^b^
Patient assessment and problem tracking	Provide a synthetic presentation of the patient Provide guidance on the reasoning behind patient assessment Assist in problem recognition Select indicators based on the patient’s problem	Improve access to vital signs trends and displayed graphs Distinguish chronic and acute problems	Optimize access and display of relevant information based on patient condition	Provide an overview with targeted information based on the patient’s problem
Clinical indicator monitoring, and notification and alert optimization	Combine relevant data from different sources (eg, laboratory results, physiological parameters, and monitoring) Provide reminders of target values for the clinical indicators, and alert when abnormal values are reached Integrate measurements and data collected by nurses	Highlight important information (eg, abnormal values, reminders, and new results notifications)	Adapt notifications for quick and easy interpretation	—
Access and adherence to clinical practice guidelines	Monitor guideline adherence Alert when actions do not align with the best recommendations.	Integrate recommendations into prescriptions	Harmonize and facilitate access to evidence-based references	Incorporate practice support procedures
Integration of decision support algorithms	Diagnostic aid Ventilatory weaning Vasopressor weaning	Prescription aid Transfusion	Prediction of patient deterioration	Automating standard prescriptions (eg, change of route and medication)

^a^PICU: pediatric intensive care unit.

^b^Data not available.

##### Patient Prioritization

Clinicians expressed a need to prioritize patients according to the severity of their condition and to quickly detect any changes. A participant mentioned the value of rapid assessment of the patient status as follows:

...give me a quick view, actually, of whether a patient is stable vs. not stable, or critical, or an alert for a change in situation.

To help clinicians prioritize patients during handoff meetings or medical rounds, we aimed to optimize patients’ visualization with a user-friendly, interactive, and customizable display while adding stability indexes according to the patients’ conditions.

##### Patient Assessment and Problem Tracking

- Patient portrait: Clinicians were looking for a synthetic presentation of patient’s data to optimize clinical assessment, as expressed by 1 participant as follows:

If we would be able to make a patient Dashboard with a synthetic presentation of the different elements. Passing some of the elements that the electronic record should do to us, but that doesn’t do too much and that considers the temporality, that considers these important clinical elements and that are in real time, or at least close.

- Problem monitoring: Clinicians must recognize patient problems to guide and facilitate data analysis and monitor patient outcomes. This was highlighted by a participant as follows:

...help me more finely, when I’m on rounds or when I’m assessing a patient, help me in my reasoning or in my diagnosis or in my assessment of the patient. At that time, to have a more accurate view of the patient’s condition, for conditions that are complex.

Participants highlighted the importance of monitoring patients according to their condition and early detection of problems, as follows:

(...)Depending on the pathology of the patient, it would be nice if it was the thing that detects it on its own, if the patient has respiratory distress.

Our goal was to optimize the evaluation of the patient’s current state through a synthetic presentation by selecting relevant clinical information for the patient’s assessment and improving the patient’s progress monitoring in intensive care. The complete clinical data remained accessible in the EHRs for detailed analysis.

##### Clinical Indicators Monitoring and Notification or Alert Optimization

Although EHRs enable monitoring of clinical indicators across several sections, including medical and nursing progress notes, clinicians believed that notes could be optimized with automatic data extraction and updates. One participant stated as follows:

Notes are worth what they are worth, there are people who write good notes, there are people who don’t write good notes. I think there's a lot of copy-paste. So sometimes you go back into the notes and you’re going to see the exact same thing at 5 days online in different sections. It's the conclusion that changes a little bit.

Alerts were also crucial for early detection of changes in the patient’s disease course. One participant said:

It helps, the little logos come out quicker. It helps identify what's more abnormal, more quickly.

The goal was to monitor indicators according to the body system or problems associated with the system, maintaining the same structure used in presenting and assessing patients during clinical activities and within the EHR. Customizing notifications facilitated data processing and intervention planning. This could be achieved by opting for simple color codes, avoiding intrusive alerts, and duplicating information.

##### Access to Evidence-Based Recommendations

Clinicians must incorporate evidence-based recommendations and guidelines into their decision-making processes. Easy and standardized access to scientific databases is essential. One participant explained the need for clinical practice guidance as follows:

I think we could do better, to see the number of variations for a problem is that there’s not a lot of scientific rigor. Could this compensate for things that are done by repetition, by reflex without foundation, and that it would be more supervised or with a better scientific basis? Probably.

The aim was to optimize access to clinical practice guidelines either by integrating the recommendations directly into data analysis through reminders and suggestions or providing direct access to scientific databases.

##### Integration of Decision Support Algorithms

This involved development work to associate knowledge bases with patient data using inference engines or artificial intelligence algorithms. Prediction of patient condition deterioration, diagnosis support, prescription support, and sedation weaning were among the main expressed needs.

These algorithms aimed to guide clinical decision-making. Their integration could be performed at diagnostic, therapeutic, and preventive levels. This study aimed to provide a structure susceptible to accommodating new algorithms and decision support features. Therefore, according to the first reflections, we intended to associate these algorithms with body systems or the patient’s problems.

### Data Structure

**Figure 3:**
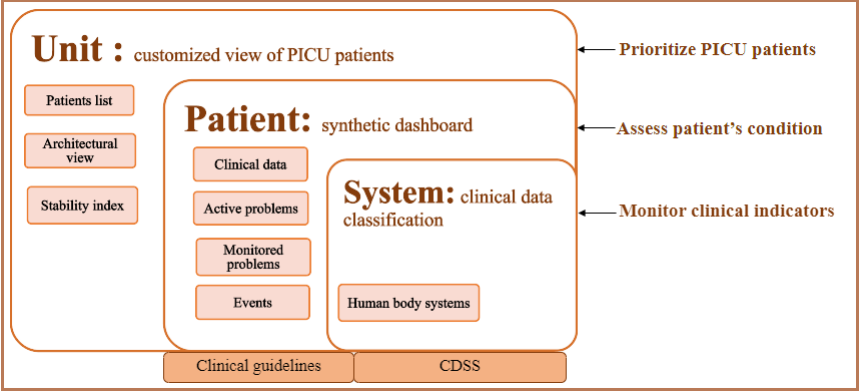
The resulting structure for data representation in the pediatric intensive care unit (PICU) with 3 levels of abstraction. CDSS: clinical decision support system.

To structure emerging elements while adapting data representation to the clinical workflow, we opted for a 3-level system structure (Figure 3). These levels mainly reflect the first 3 identified themes, whereas the themes related to clinical practice guidelines (#4) and decision support algorithms (#5) can be attached to different levels, depending on the context of use and the problem being addressed.

1. *Unit level (patient prioritization):* The first level provided a global view of patients in the PICU, with various display modes allowing efficient management of patient lists and easy identification of those who were unstable and helping to plan bedside activities. Adding stability indices helped to categorize and prioritize patients based on their condition severity.
2. *Patient level (patient assessment):* For each patient, clinicians could access this second level to obtain a quick overview of the patient’s condition and better understand the underlying cause of their instability. The patient’s synthetic presentation allowed clinicians to assess the patient’s status based on current problems and probable complications and to track important events. Eventually, incorporating guidance and evidence-based recommendations would be pertinent at the second and third levels.
3. *System level (indicator monitoring):* The third level was intended to align with the clinical flow by assessing patients according to their body systems (eg, neurological, respiratory, cardiovascular, renal, gastrointestinal, hematological, immunologic, infectious, and musculoskeletal tissue systems). This allowed monitoring of the degree of alteration of the system based on associated clinical indicators. Moreover, this level aimed to integrate clinical decision support algorithms developed in response to specific problems (eg, evaluation of head trauma management associated with the neurological system).

### Prototype Design

#### Overview

Using the defined structure for clinical data representation, we designed interfaces corresponding to the 3 levels of the structure. Subsequently, design meetings allowed us to adapt the design and integrate, early in the process, the necessary adjustments to meet the end users’ needs. This section presents the design and adjustments of the prototype. Each interface included a targeted functionality in response to the objectives of the associated level.

We used a brain injury care scenario to illustrate the functioning model of the preliminary prototype, knowing that a patient with a severe traumatic brain injury requires attentive monitoring that involves clinical data from different devices (eg, mean arterial pressure, oxygen saturation, temperature, end-tidal carbon dioxide, and brain tissue oxygenation), laboratory results, ongoing sedation, and medication treatment, along with the interpretation of imaging examinations. To assess the patient’s status and, therefore, adjust their care plan, clinicians should be able to categorize the patient according to a severity scale, identify whether there is a risk of deterioration (eg, ischemia and hyperemia), define optimal mean arterial pressure in the context of brain injury, and quickly recognize abnormal values depending on the patient’s profile. We expected that this would help clinicians to focus on pertinent details and support the prediction of changes in the patient’s condition.

#### Unit Level

The objective of this level was to visualize all inpatients in 2 display modes and introduce the concepts of stability and system alteration.

**Figure 4:**
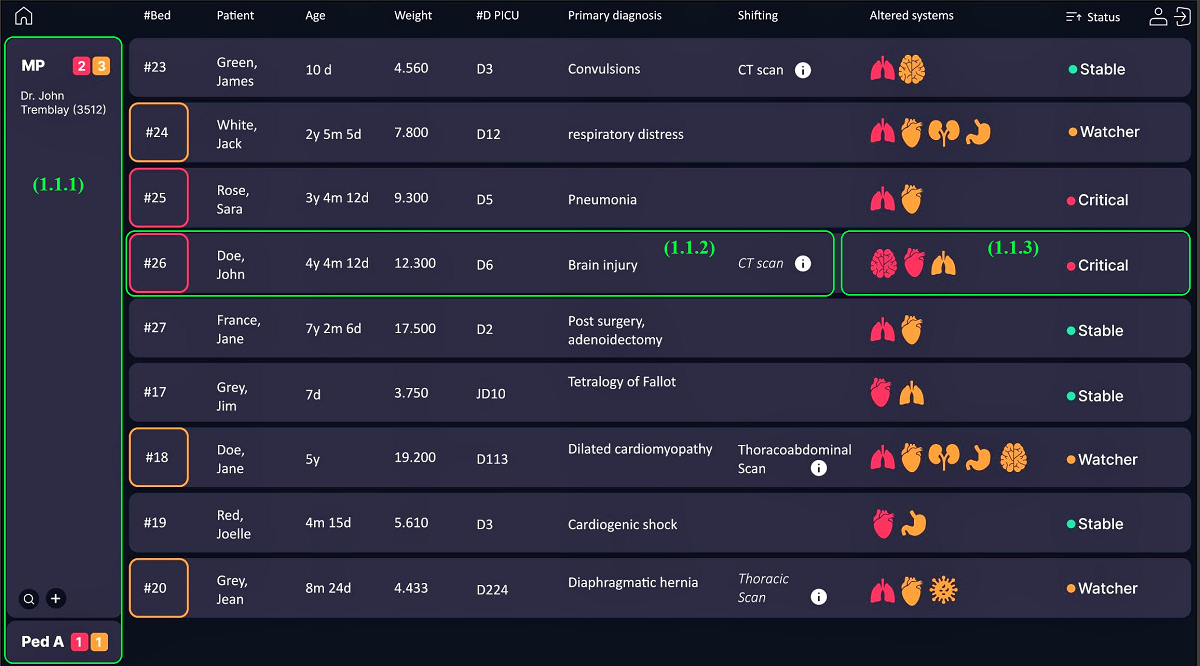
Level 1 interface in the preliminary prototype: patients list. This figure includes lists management (1.1.1), patient identification (1.1.2), and stability indices (1.1.3).

The list-mode display (Figure 4) allowed clinicians to select patients by service (Pediatric A, B, or C) or to create their personalized list (My Patients) by adding patients under their responsibility. Every patient on the list was identified with a bed number, name, age, weight, length of stay, and diagnosis. It also included scheduled tests or procedures that required off-unit transportation to assist the clinician in scheduling bedside interventions. Stability indices were added to help clinicians prioritize patients and plan their interventions for the day. These indices included a list of altered body systems and the status to categorize patients according to their conditions: critical, watcher, stable, or discharged. We used red alerts to indicate a severe alteration or criticality level and orange alerts to indicate a moderate level.

**Figure 5:**
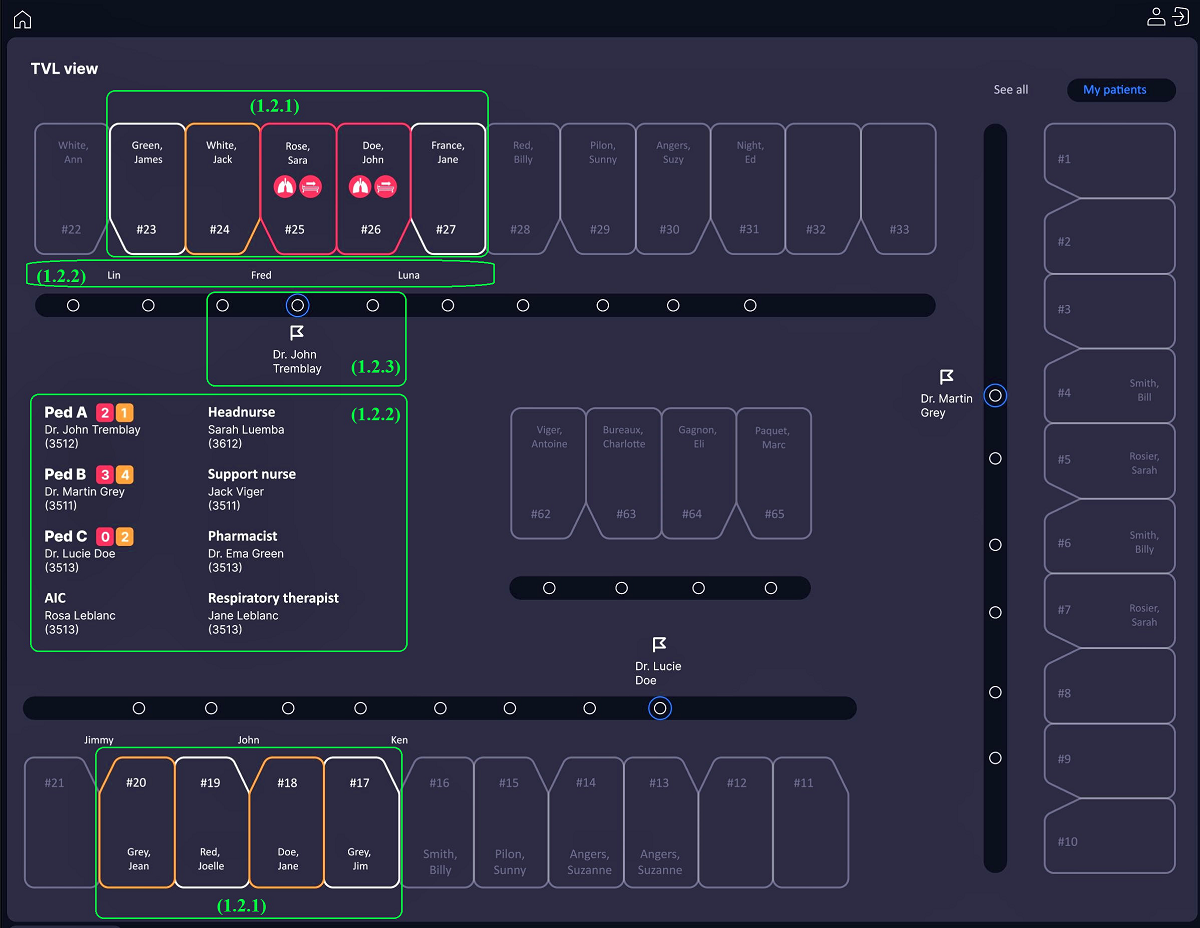
Level 1 interface in the preliminary prototype: unit architectural view. This figure includes color-coded boxes for patients according to their stability (1.2.1), the team in charge (1.2.2), medical team location (1.2.3).

Furthermore, level 1 provided an architectural view of the unit (Figure 5) inspired by the TVL, which was adapted to assist clinicians in planning medical rounds and bedside interventions. Clinicians could customize the display to view their patients or all inpatients.

The stability indicators display observed the same color code for the boxes: red for the critically ill and orange for less acuity. This view allowed us to see the list of available caregivers with their contact numbers and the nurses responsible for the patient’s bedside. Notably, geolocation of the team’s location during medical rounds could help plan clinical interventions. For example, a clinician who needed to join the medical round for a specific patient could check this interface to plan his or her next tasks to match the team’s arrival at the patient’s bedside.

To assess a specific patient, clinicians could select the patient from the patient list or switch to the TVL view and access synthetic data presentation at the second level.

#### Patient Level

**Figure 6:**
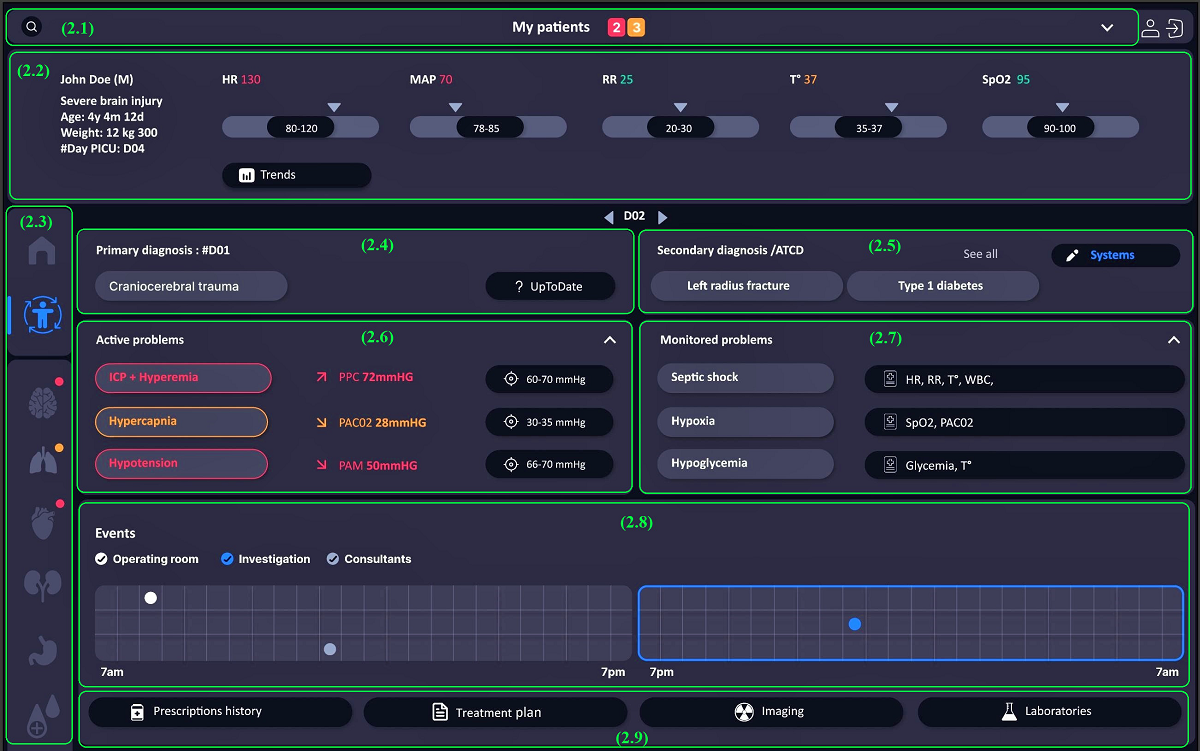
Level 2 interface in the preliminary prototype. In this figure, we focused level 2 on a brain injury case scenario. The features include access to personal list (2.1), patient identification and vital signs (2.2), navigation menu (2.3), primary and secondary diagnosis (2.4 and 2.5), patient’s active problems (2.6), problems under surveillance (2.7), important events (2.8), and access to clinical systems (2.9).

Continuous monitoring of inpatient progress was central to clinical activities in the PICU. Therefore, the patient level (Figure 6) was incorporated into the prototype design to facilitate the evaluation of the patient status and progress during their stay in the unit. The second level provided an overview of the patient’s active problems and likely risks based on monitoring relevant indicators.

Clinicians could easily return to their personalized lists and search for patients. Color-coded notifications indicated the number of critical patients (red) and watcher patients (orange). The patient identification zone included demographic data, initial diagnosis, and length of stay in the unit. The same zone displayed vital signs in real values, with possible access to trends observed in the last few hours. The left menu allowed quick navigation between levels 1 and 2 and through the body systems at level 3. This interface provided information about the patient’s primary diagnosis, with the last revision date, and allowed clinicians to access a direct link to the UpToDate (Wolters Kluwer) knowledge base [45], which was widely used for medical decision support. Secondary pathologies and patient history were also listed and allowed sorting by body systems. Furthermore, active problems were displayed and an interpretation of abnormal indicator values were provided, with reminders for target values, to facilitate the recognition of the patient’s problems. Finally, problems under surveillance were shown to guide clinicians in patient care by targeting probable complications. Decision support systems could be incorporated into this level and connected to a patient’s problem. For example, a patient with respiratory failure (a medical problem) could have a CDSS for the early diagnosis of acute respiratory distress syndrome [36] and another for the management of mechanical ventilation if diagnosed with acute respiratory distress syndrome [46]. This level allowed the tracking of significant events, such as procedures performed in the operating room, specific investigations, and consultant visits. When needed, clinicians could search for additional information by directly accessing clinical applications and systems, which could be related to prescription history, treatment plans, or recent imaging or laboratory tests.

Clinicians could visualize clinical indicators on the third level to closely monitor these indicators related to patient problems (refer to *system level* section).

#### System Level

**Figure 7:**
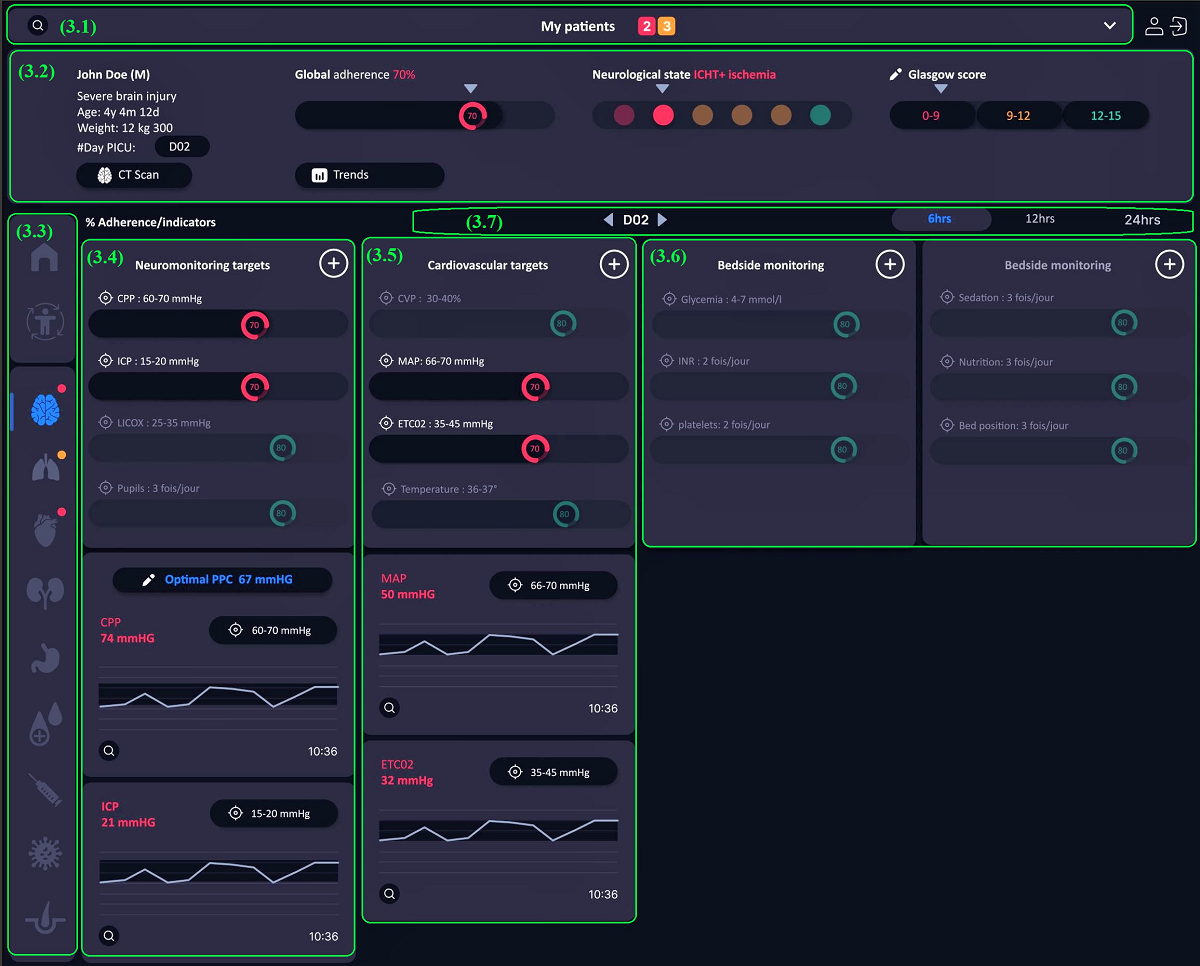
Level 3 interface in the preliminary prototype. The model includes access to personal list (3.1), patient identification and system global indicators (3.2), navigation menu (3.3), system indicators (3.4), problem-related indicators from other systems (3.5), additional indicators (3.6), and indicators evolution (3.7).

The third level (Figure 7) was designed to display groups of indicators related to human body systems and to access decision support tools developed for specific problems involving these systems. Our goal was to prioritize indicators to be monitored based on body system alterations while retaining the ability to add indicators from other systems to refine clinical decisions. In Figure 7, we included indicators of the brain injury care scenario and added a visualization tool to assess clinician adherence to the clinical practice guidelines. The development of a visualization tool will be subject to further research.

The personalized patient list could also be accessed at this level. The patient identification zone included demographic data, initial diagnosis, length of stay in the unit, and data related to patient progress in the care trajectory. For a head injury, clinicians could assess the global adherence to clinical practice guidelines and follow, through trends, changes in the patient’s neurological status and Glasgow score. This zone also provided access to the last computerized tomography scan performed. As in the previous level, the navigation menu allowed users to browse between different levels and different systems at the third level. Altered systems were easily identified using simple color-coded signs (eg, red for highly severe indicators and orange for less severe indicators). The first group contained specific indicators related to the neurological system; this area allowed clinicians to evaluate adherence to guidelines for brain injury indicator monitoring and management. Abnormal values were systematically displayed, with access provided to trends observed in the last few hours. Furthermore, clinicians could view trends in normal indicators or add other neurological indicators not directly related to head injury care. The interface also allowed users to view indicators belonging to other systems but related to the patient’s problem. For example, surveillance of a patient with a head injury is not limited to neurological indicators but covers variable indicators, such as cardiovascular and respiratory indicators. Other groups were included for bedside monitoring. In addition, the interface enabled data display by date or time range to optimize clinical indicator monitoring.

## Discussion

### Principal Findings

In this study, we took the first steps to develop a decision support structure that responds to clinician needs in the PICU. We analyzed the existing situation to evaluate current needs, which led us to develop a 3-level data representation structure, with the first level aimed at prioritizing inpatients based on the severity of their conditions, the second level providing an overview of the patient’s condition and evolution, and the third level allowing close monitoring of clinical indicators related to a specific problem or human body system. From this perspective, the third level was intended to support CDSS integration as developed in response to specific care management needs related to the patient’s condition. In subsequent steps, there will be a testing process involving end users to validate the usability and performance of the designed prototype.

Our goal was to create a system based on the proposed representation and eventual CDSS integration. It is important to note that this system is not intended to replace EHRs designed for documenting patient care or any other existing systems. However, its use should help clinicians prioritize their interventions according to patient’s needs, which could be applied to the handoff meetings while discussing inpatient conditions and planning next-shift interventions. Furthermore, the tool could optimize clinicians’ cognitive processes by readily accessing relevant information when needed, such as for patient presentation during medical rounds, for fast checks on patient status and detection of any changes. In addition, the display of the clinical indicators could be personalized to suit the user’s preference and optimize clinical monitoring by allowing an adequate and efficient classification of indicators either by the human body system or by patient problem, which helps to contextualize data evolution.

### Limitations

Although the features presented in the Principal Findings Section are generally appreciated by the clinicians, they, nonetheless, remain prudent regarding the following concepts. The first concept is patient criticality assessment, knowing that criticality could be linked to variable factors, such as a combination of a patient physiological profile, care required, and intensity of that care [47], perception related to patient prognosis, illness progression and response to treatment [48], and severity scores used to measure deviations observed in certain groups of physiological variables [49]. The second concept is problem progression, which could be difficult to track because information at the start and end of a problem is not always accurate. Although a change is usually identified by a deviation from normal or expected values, it ultimately depends on the patient’s progress in their care trajectory [50]. The third concept is that some problems affect multiple body systems and certain specific indicators related to such problems [51]. This requires classifying the indicators by problem and defining abnormal variations for each indicator according to the patient’s physiological and pathological profiles. For example, the mean arterial pressure indicator is related to the cardiovascular system, but for a patient with a head injury, this indicator directly affects cerebral perfusion; therefore, its monitoring is also linked to the neurological system. Furthermore, the thresholds for this indicator may vary with the patient’s age and illness history.

Clinical judgment is crucial for patient assessment and decision-making in critical care. This judgment varies among clinicians and relies on each clinician’s ability to synthesize relevant clinical data, which is not easy to model.

Analyses of these factors will eventually help us to optimize our data representation model in terms of the connections between problems and human body systems. In addition, identifying the factors that influence the progression of problems will help in predicting the deterioration of a patient’s condition and preparing an appropriate intervention.

In our study, we initially envisaged a sample of 30 participants (6 physicians, 4 fellows, 3 residents, 2 specialized nurse practitioners, and 15 nurses) to have a better representation of the targeted population. However, the desired sample was not achieved owing to the limited availability of PICU staff and their high workload during the project period, which was during the Covid-19 pandemic. A total of 11 participants could participate in the interviews. Only 5 (45%) physicians participated in the design meetings during the second phase of the project.

Through the design meetings, we could improve the prototype design. However, we were unable to test the final version with the clinicians. Therefore, we intend to conduct usability tests afterward to identify potential issues and ensure that end users are satisfied with the resulting prototype. Future work should also investigate the integration of the prototype into the clinician’s workflow. Although the prototype intended to synthesize relevant clinical data from other sources into a consistent view, it could increase the clinician’s workload by adding another technological tool to consult the patient’s condition. This requires careful consideration of the tools’ interoperability to follow the clinician’s role.

### Conclusions

This study provided a clinical data representation structure to support PICU clinicians in their decision-making process and to assist them in optimizing inpatient care management.

An observation of clinical activities and interviews with participants allowed us to identify the current needs for decision support. Through an analysis of collected data, we created a structure with 3 levels of abstraction to facilitate patient prioritization, assessment, and monitoring. A prototype was designed based on the main structure and then presented to the participants to obtain feedback for improvement.

Notably, the functionalities integrated into the prototype mainly met the clinicians’ expectations regarding information relevance and classification. Adjustments were made to the data representation following the design meetings with the participants. However, further tests will be conducted to ensure the tool’s usability.

To enable the deployment of the proposed decision support structure and its integration into the clinical workflow in the PICU, further analysis and development are needed to establish patient stability indices, automate problem recognition, and define the indicators associated with each body system and the respective alteration thresholds.

## Abbreviations

CDSS: clinical decision support system
EHR: electronic health record
PICU: pediatric intensive care unit
TVL: Tableau de visualisation de lits (beds visualization table)

## Data Availability

Data sharing is not applicable to this study as no data sets were generated or analyzed during this study.
